# Bibliography

**Published:** 1886-07

**Authors:** 


### BIBLIOGRAPHY.

MIND YOUR EYES! Good advice from a near-sighted man to his fellow-sufferers; translated from the French of Francisque Sarcey, by Henry Dickson Bruns, M. D., visiting Oculist to the Charity Hospital, New Orleans. N. O., published by the N. O. Medical Publishing Association, 1886.

This is a neat little "booklet," as the *N. C. Medical Journal* calls it; being neither book nor pamphlet—and is written in that sprightly, piquant, vivacious style so eminently "Frenchy." It is captivating,—and once taken in hand, one cannot lay it down until it is finished. Dr. Bruns very modestly says, that "if he can't write a book he can translate one that somebody else has written." In this he has conferred a boon on suffering near-sighted people, and a real pleasure on those who will not "mind their eyes"—but will read,—the omniverous reader. An idea of the style and contents of this refreshing little *morceau* may best be gotten from an extract;—and what more appropriate extract can we give than a "cataract extract-" ion?

"The doctor said to me: 'Keep looking down, and take things easy; don't strain on any account.' What easier to do than this?

"'All right,' said I to myself, 'I will not strain, if that is the order.' And I strained with all my might to keep myself from straining. As to looking down, it was impossible. While I saw, for I could still see a little, the devilish knife crawling up under my upper lid, my eye turned irresistably towards it.

"'Look down,' said M. Perrin, gently; 'Look down,' said Felizet in what was meant to be a firm tone.

As for me, I felt for myself mingled contempt and anger, for every time that, by a tremendous effort of the will, I turned my eye down, it got away again and flew upward as if worked by a spring.

"'I am ashamed of myself, doctor,' said I, in a most extraordinary state of weakness. 'I am mortified, humiliated; I thought that one was always master of his own movements; I am not; it is mortifying. What is the use of being a philosopher?"

"'Calm yourself,' said M. Perrin; 'these are reflex movements.' * * * * * * *

Taking advantage of a movement during this conversation, M. Perrin had opened the eye, and the lens had escaped; the operation was done, and well done, with a swiftness and certainty of which Felizet still speaks with admiration.

"'Do you see my hand,' said the operator, shaking it before my eye. I saw it through a sort of fog. 'Yes, I see it,' I said. 'Good, it's all over.'"

Now—is'nt that style refreshing?—(to the reader, not to the patient). Write to Wharton Bros., 5 Carondelet street, New

Orleans, for a copy—price 50 cents; and mention this journal, please.

---

The Principles and Practice of Surgery, by Frank Hastings Hamilton, A. M., M. D., LL. D., late Professor of the Practic of Surgery; with Operations, and of Clinical Surgery, in Bellevue Hospital Medical College; Consulting Surgeon to Bellevue Hospital; to the Bureau of Surgical and Medical Relief for the Outdoor Poor, at Bellevue Hospital; to St. Elizabeth's Hospital, and to the Hospital for the Ruptured and Crippled; Fellow of the New York Academy of Medicine, etc. Illustrated with 472 engravings on wood. Third edition, revised and corrected. New York, Wm. Wood & Co.

The fisst edition of this celebrated work was issued in 1872. It became very popular at once and was soon a standard in the schools. The work, we may say, represents the conclusions arrived at as to the best practical proceedure in surgery after a long experience in teaching, and in the practice of surgery both in civil and military life, by this author. It is not designed, therefore, as a complete treatise on surgery, but as a "text book for students, and a direct and complete *guide*" in surgical practice, according to the author's own views of the subject. A second edition was issued in 1873, in which no material changes were made;—typographical errors were eliminated, certain statements modified and a few important facts were added only.

But since that time such progress has been made—almost a revolution, it may be said, having taken place in opinion, since the introduction of antiseptics, that it became necessary to greatly modify a portion of the text, while the original scope and intentions have been rigidly adhered to, not that Dr. Hamilton has adopted all that is claimed for antiseptics or "Listerism," for, we believe, he is skeptical as to the "germ theory," —but, as he says—in the preface to the third edition:

"To one whose experience has been obtained while these changes were in progress [the introduction of anæsthesia in his early professional life, and that of antiseptics in his later life] —a period so brief in point of time, but so widely separated in their practices, pretences and hopes, it will be granted to institute comparisons, and to draw conclusions. This privilege has been exercised whenever a suitable opportunity has been presented."

The third edition is gotten out by Wood & Co., in unusually substantial and handsome style.

---

A Doctor's Experiences in Three Continents. By Edward Warren—Bey, M. D., C. M., LL. D. Cushing & Bailey, publishers. Baltimore, Md.: 1885; 12mo.; pp. 613; cloth.

This volume is made up of a series of thirty-five letters, addressed to John Morris, M. D., Baltimore, Md.

In the first eight letters Dr. Warren gives a brief history of his family, of his Scottish ancestry, of his early life, and college days, and intersperses it with many episodes which befall boys, who, in mature years, become authors.

In the next eight letters, he gives a history of his visit to Paris in 1854, and his return to North Carolina, the State of his nativity, and of the work and incidents of his life until the commencement of hostilities between the Confederate States and the United States in 1861.

In ten letters he gives an interesting and graphic description of his connection with the Southern cause, and of the memorable transactions and scenes during those years,

In 1872 Dr. Warren visited Egypt, and in the remaining nine letters he gives us a brief and instructive account of that people and their customs, and of his service in the army of the Khedive.

The style of the author is that of a narrative, giving the scenes, incidents, and history of the hero in all the important details of his life; and be assured the author has not left the reader to conjecture who the hero is. But the work is a valuable contribution in its historical details, and will well repay the reader for the time consumed in reading it.

---

METHODS OF BACTERIOLOGICAL INVESTIGATION. By Ferdinand Heuppe, Docent in Hygiene and Bacteriology in the Chemical Laboratory of R. Fresenius, at Weisbaden. Translated by Herman M. Biggs, M. D., Instructor in Carnegie Laboratory, and assistant to the Chair of Pathological Anatomy in Bellevue Hospital Medical College. Illustrated by thirty-one wood cuts. New York: 1886. D. Appleton & Son, 1, 3, 5, Bond street. Cloth; pp. 218.

Now, gentlemen, here is the book we have been looking to make its appearance to fill a long-felt want (sure enough), for you all know, and we know, that nine-tenths of the best and most experienced of practititioners know absolutely nothing about bacteria; they do not know, we will venture, the difference between a micrococcus and a bacterium, and could not tell a schizomycete from a spore. Here is a book that begins at the beginning, and tells you all about the things, what they are called, where and how to find them, how to know them when you see them, how to distinguish one from another, and how to propagate them, how to stain and mount them, and all about it; just like taking a child and beginning with A, B, C, and a-b, ab, and so forth. Moreover, the book is written in plain and simple language, and is easily understood. The classification is so simple that a child could understand it. Now that the germ theory of disease is generally accepted, and bacteria enter so largely into the pathogenesis of many ailments; now that bacteriology has become a legitimate study, as essential as pharmacology or any other ology, every physician should inform himself of at least the rudiments of the subject. In fact, he must do so, or he can never follow the developments that research are daily opening up before us. Truly, the miscroscope has opened up another world, of whose existence our grandfathers had never dreamed. This book will teach you how to go to work if you have any desire to experiment with the denizens of that microcosm—those poisonous little fellows at whose door so many sins are laid. It is gotten up in Appleton's usual neat style.

THE STUDENTS MANUAL OF VENEREAL DISESES. Being a concise description of those affections and of their treatment. By Berkeley Hill, M. D., Professor of Clinical Surgery in University College, London. Surgeon to University College and the Lock Hospitals; and Arthur Cooper, M. D., Surgeon to the Westminter General Dispensary : formerly House Surgeon to the Lock Hospital. Fourth edition. Revised. Philadelphia: P. Blakiston Sons & Co., 1012 Walnut str. 1886. Cloth . pp. 132.

This is a valuable little work—a sum of many of the latest teachings and experiences with that intractable class of diseases which every general practitioner encounters daily. Should be read by all practitioners and especially will it be of assistance to the beginners.

---

AN ANALYSIS OF THE URINE, with Special Reference to the Diseases of the Genito-Urinary Organs, by K. B. Hoffmann, Professor in the University of Gratz, and R. Ultzmann, Docent in the University of Vienna. Translated by T. Barton Brune, A. M., M. D., late Professor of the Practice of Medicine in the Baltimore Polyclinic and Post-Graduate Medical School, and Lecturer on Clinical Medicine University of Maryland; and H. Holbrook Cu tus; Ph. B. M. D., Fellow New York Academy of Medicine, etc.
Second edition, revised and enlarged, New York, D. Appleton & Co., 1, 3. 5 Bond street, 1886. Cloth, pages 310.

This very valuable and very practical book contains sixteen handsome chromo-lithographs of microscopic appearance of epithelium from kindey tubes, ditto from male and female urethra. Primary forms of uric acid crystals (the so-called whetstone crystals), uric acid as found in "native urine," nitrate of urea, triple phosphates, leucine and tyrocine, the sediment of alkaline fermentation, hæmin, urate of sodium, cystine, calcium carbonate, (very rare), hyaline, granular and waxy cylinders, pus corpuscles, yeast fungi, cancer elements, etc., etc., not mentioned in the title page.

The best criticism we can make on this work is to quote the words of the translator, in his preface: "It will supply a need which has been long felt by American students and physicians. We do not know of a single work in the English language, where, in a concise form, so many valuable suggestions and practical hints are offered, both as regards analysis and diagnosis."

The book is turned out in the neat and substantial form characteristic of the great house of Appleton. Their books fairly illuminate ones library.

